# Existence and Hadamard well-posedness of a system of simultaneous generalized vector quasi-equilibrium problems

**DOI:** 10.1186/s13660-017-1330-2

**Published:** 2017-03-07

**Authors:** Wenyan Zhang, Jing Zeng

**Affiliations:** 1 Department of Economic Mathematics, Southwestern University of Finance and Economics, Chengdu, 610074 China; 2 College of Mathematics and Statistics, Chongqing Technology and Business University, Chongqing, 400067 China

**Keywords:** 49J53, 49K40, 90C33, 90C46, demicontinuity, natural quasi-convexity, existence theorem, Hadamard well-posedness

## Abstract

An existence result for the solution set of a system of simultaneous generalized vector quasi-equilibrium problems (for short, (SSGVQEP)) is obtained, which improves Theorem 3.1 of the work of Ansari et al. (J. Optim. Theory Appl. 127:27-44, 2005). Moreover, a definition of Hadamard-type well-posedness for (SSGVQEP) is introduced and sufficient conditions for Hadamard well-posedness of (SSGVQEP) are established.

## Introduction

Recently, a vector equilibrium problem has received lots of attention because it unifies several classes of problems, for instance, vector variational inequality problems, vector optimization problems, vector saddle point problems and vector complementarity problems, for details, see [2] and the references therein. Moreover, many authors further investigated several general types of it, for instance, see [3–8].

Let *I* be a finite index set and $i\in I$. Assume that $E_{i}$, $F_{i}$ and $Z_{i}$ are locally convex Hausdorff spaces, $X_{i}\subset E_{i}$ and $Y_{i}\subset F_{i}$ are two nonempty convex subsets. Let $X=\prod _{i\in I}X_{i}$ and $Y=\prod _{i\in I}Y_{i}$. Assume that $C_{i}:X\rightarrow2^{Z_{i}}$ is a set-valued mapping, the values of which are closed convex cones with apex at the origin, $C_{i}(x)\subsetneqq Z_{i}$ and $\operatorname {int}C_{i}(x)\neq\emptyset$. Let $Z_{i}^{*}$ be the dual of $Z_{i}$, $S_{i}: X\rightarrow2^{X_{i}}$ and $T_{i}: X\rightarrow2^{Y_{i}}$ be set-valued mappings with nonempty values. Assume that $f_{i}:X\times Y\times X_{i}\rightarrow Z_{i}$, $g_{i}: X\times Y\times Y_{i}\rightarrow Z_{i}$ are two trifunctions.

One of the general types, a system of simultaneous generalized vector quasi-equilibrium problems (for short, (SSGVQEP)), as follows, is considered: find $(\bar{x},\bar{y})\in X\times Y$ such that $\forall i\in I$, $\bar{x}_{i}\in S_{i}(\bar{x})$, $\bar{y}_{i}\in T_{i}(\bar{x})$,

$$\begin{aligned}& f_{i}(\bar{x},\bar{y},u_{i})\in C_{i}(\bar{x}),\quad \forall u_{i}\in S_{i}(\bar{x}), \\ & g_{i}(\bar{x},\bar{y},v_{i})\in C_{i}(\bar{x}),\quad \forall v_{i}\in T_{i}(\bar{x}). \end{aligned}$$

The problem (SSGVQEP) was introduced by Ansari in [1]. By suitable choices of $f_{i}$, $g_{i}$, $S_{i}$ and $T_{i}$, (SSGVQEP) reduces to several classical systems of (quasi-)equilibrium problems and systems of variational inequalities, which are studied in the literatures (see [9–13] and the references therein). Furthermore, by suitable conditions and suitable choices of *i*, (SSGVQEP) contains vector equilibrium problems as special cases. A solution of (SSGVQEP) is an ideal solution. It is better than other solutions such as weak efficient solutions, efficient solutions and proper efficient solutions (see [2, 14–16] and the references therein). Therefore, it is meaningful to study the existence result for the solution set of (SSGVQEP).

The classical concept of Hadamard well-posedness requires not only the existence and uniqueness of the optimal solution but also the continuous dependence of the optimal solution on the problem data. Recently, the classical concept together with its generalized types has been studied in other more complicated situations such as scalar optimization problems, vector optimization problems, nonlinear optimal control problems, and so on, see [4, 17–29] and the references therein. However, as far as we know, there are few results about Hadamard well-posedness of (SSGVQEP). Therefore, it is necessary to study Hadamard well-posedness of (SSGVQEP).

In this paper, by using demicontinuity and natural quasi-convexity, we obtain an existence theorem of solutions for (SSGVQEP). Moreover, we introduce the definition of Hadamard well-posedness for (SSGVQEP) and discuss sufficient conditions for Hadamard well-posedness of (SSGVQEP). The rest of the paper goes as follows. In Section 2, we recall some necessary notations and definitions. In Section 3, we obtain the existence theorem of solutions for (SSGVQEP). In Section 4, we investigate Hadamard well-posedness of (SSGVQEP).

## Preliminaries and notations

Let us recall some notations and definitions of vector-valued mappings and set-valued mappings together with their properties.

Let *X*, *Y* be two topological spaces and $F:X\rightarrow2^{Y}$ be a set-valued mapping. Assume that $x\in X$. If for any open set *V* with $F(x)\subset V$, there exists a neighborhood *N* of *x* such that

$$\bigcup_{x'\in N}F\bigl(x'\bigr):=F(N) \subset V, $$

*F* is called upper semi-continuous ($\mathit{u.s.c.}$ for short) at *x*. If *F* is $\mathit{u.s.c.}$ at each point of *X*, *F* is called $\mathit{u.s.c.}$ If for any $z\in F(x)$ and any neighborhood *N* of *z*, there exists a neighborhood *U* of *x* such that $\forall y\in U$, we have

$$F(y)\cap N\neq\emptyset, $$

*F* is called lower semi-continuous ($\mathit{l.s.c.}$ for short) at *x*. If *F* is $\mathit{l.s.c.}$ at every point of *X*, *F* is called $\mathit{l.s.c.}$ In addition, *F* is called continuous if *F* is both $\mathit{l.s.c.}$ and $\mathit{u.s.c.}$ If the set $\operatorname{Graph}(F)$, i.e., $\operatorname{Graph}(F)=\{(x,y):x\in X,y\in F(x)\}$, is a closed set in $X\times Y$, *F* is called a closed mapping. *F* is called compact if the closure of $F(X)$, i.e., $\overline {F(X)}$, is compact, where $F(X)=\bigcup_{x\in X}F(x)$.

### Definition 1

[30]

Let *Y*, *Z* be topological vector spaces. A vector-valued mapping $f:Y\rightarrow Z$ is called demicontinuous if for each closed half space $M\subset Z$,

$$f^{-1}(M)=\bigl\{ x\in Y: f(x)\in M\bigr\} $$

is closed in *Y*.

### Definition 2

Let $(Z,P)$ be an ordered topological vector space, *E* be a nonempty convex subset of a vector space *X*, and $f: E\rightarrow Z$ be a vector-valued mapping.

(i) *f* is called convex if for every $x_{1},x_{2}\in E$ and for every $\lambda\in[0,1]$, one has
  $$f\bigl(\lambda x_{1}+(1-\lambda)x_{2}\bigr)\in\lambda f(x_{1})+(1-\lambda)f(x_{2})-P. $$
(ii) *f* is called properly quasi-convex if for every $x_{1},x_{2}\in E$ and $\lambda\in[0,1]$, one has either $f(\lambda x_{1}+(1-\lambda)x_{2})\in f(x_{1})-P$ or $f(\lambda x_{1}+(1-\lambda )x_{2})\in f(x_{2})-P$.
(iii) *f* is said to be naturally quasi-convex if for every $x_{1}, x_{2}\in E$, $\lambda\in[0, 1]$, there exists $\mu\in[0, 1]$ such that
  $$f\bigl(\lambda x_{1}+(1-\lambda)x_{2}\bigr)\in\mu f(x_{1})+ (1-\mu)f(x_{2})-P. $$

It is clear that every properly quasi-convex or convex mapping is naturally quasi-convex, but a naturally quasi-convex mapping may not be convex or properly quasi-convex.

## Results and discussion

In this section, we will consider the existence results of (SSGVQEP) and give an example to show that our existence theorem extends the corresponding result in [1]. Moreover, we will introduce Hadamard-type well-posedness for (SSGVQEP) and establish sufficient conditions of Hadamard-type well-posedness for (SSGVQEP).

### Existence of solutions for (SSGVQEP)

In this subsection, we will consider the existence results of (SSGVQEP) and give example to show that our existence theorem extends the corresponding result in [1].

Let *Z* be a locally convex Hausdorff space, $P\subset Z$ be a closed convex and pointed cone, and $\operatorname {int}P\neq\emptyset$. We denote

$$T=\bigl\{ x^{*}\in Z^{*}: \forall x\in-\operatorname {int}P, x^{*}(x)< 0 \mbox{ and } \forall x\in P, x^{*}(x)\geq0\bigr\} . $$

We can deduce from [31], p.165, Theorem 2, that $T\neq \emptyset$.

#### Lemma 1

*For arbitrary* $x\in Z$, *if* $(x^{*},x)\geq0$ *for all* $x^{*}\in T$, *then* $x\in P$.

#### Proof

If we assume that $(x^{*},x)\geq0$ for all $x^{*}\in T$, but $x\notin P$. Let $A=\{\lambda x+(1-\lambda) p: \lambda\in (0,1), p\in-\operatorname {int}P\}$, then we have *A* is an open convex set,

1 $$ P\cap A=\emptyset\quad \mbox{and}\quad (-\operatorname {int}P)\subset A. $$

If not, there exist $y\in P$, $\lambda\in(0,1)$ and $p\in -\operatorname {int}P$ such that $y= \lambda x+(1-\lambda)p$. Thus,

$$x=\frac{1}{\lambda}\biggl(\lambda\cdot\frac{y}{\lambda }+(1-\lambda) (-p)\biggr) \in P. $$

It is a contradiction. Thus, (1) holds. By [31], p.165, Theorem 2, there exists $x^{*'}\in Z^{*}$ such that for all $y\in P$,

$$x^{*'}(y)\geq0, $$

and for all $y\in A$,

$$x^{*'}(y)< 0. $$

Then $x^{*'}(x)< 0$ and $x^{*'}\in T$. However, this contradicts the fact that $(x^{*},x)\geq0$ for all ${x^{*}\in T}$. □

The following well-known Kakutani-Fan-Glicksberg theorem is our main tool.

#### Lemma 2

[32]

*Let* *X* *be a locally convex Hausdorff space*, $E\subset X$ *be a nonempty*, *convex compact subset*. *Let* $F: E\rightarrow2^{E}$ *be u*.*s*.*c*. *with nonempty*, *closed and convex set* $F(x)$, $\forall x\in E$. *Then* *F* *has a fixed point in E*.

#### Lemma 3

[33], Theorems 6, 7

*Assume that* *X* *and* *Y* *are two locally convex Hausdorff spaces and* *X* *is also compact*. *The set*-*valued mapping* $F: X\rightarrow2^{Y}$ *is u*.*s*.*c*. *with compact values if and only if it is a closed mapping*.

#### Theorem 1

*Let* $i\in I$. *Assume that* $E_{i}$, $F_{i}$ *and* $Z_{i}$ *are locally convex Hausdorff spaces*, $X_{i}$ *and* $Y_{i}$ *are nonempty and convex subsets of* $E_{i}$ *and* $F_{i}$, *respectively*. *Let* $X=\prod _{i\in I}X_{i}$ *and* $Y=\prod _{i\in I}Y_{i}$. *The set*-*valued mappings* $S_{i}:X \rightarrow2^{X_{i}}$ *and* $T_{i}:Y\rightarrow2^{Y_{i}}$ *are compact closed mappings with nonempty and convex values*. *Assume that the following conditions hold*:

(i) $C_{i}:X\rightarrow2^{Z_{i}}$ *is a closed set*-*valued mapping*. *For arbitrary* $x\in X$, $C_{i}(x)$ *is a convex closed cone with apex at the origin*. *Assume that* $P_{i}=\bigcap_{x\in X}C_{i}(x)$,
(ii) $P_{i}^{*}$ *has a weak* ^∗^ *compact convex base* $B_{i}^{*}$ *and* $Z_{i}$ *is ordered by* $P_{i}$,
(iii) $f_{i}:X\times Y\times X_{i}\rightarrow Z_{i}$ *is a demicontinuous function such that for arbitrary* $(x,y)\in X\times Y$,
  - $0\leq_{P_{i}}f_{i}(x,y,x_{i})$,
  - *the map* $u_{i}\mapsto f_{i}(x,y,u_{i})$ *is naturally quasi*-*convex*,
(iv) $g_{i}:X\times Y\times Y_{i}\rightarrow Z_{i}$ *is a demicontinuous function such that for arbitrary* $(x,y)\in X\times Y$,
  - $0\leq_{P_{i}}g_{i}(x,y,y_{i})$,
  - *the map* $v_{i}\mapsto g_{i}(x,y,v_{i})$ *is naturally quasi*-*convex*.

*Then* (*SSGVQEP*) *has a solution* $(\bar{x},\bar{y})\in X\times Y$.

#### Proof

We denote the set-valued mapping $T_{i}: X\rightarrow 2^{Z^{*}_{i}}$ by

$$T_{i}=\bigl\{ x^{*}\in Z^{*}: \forall x\in- \operatorname {int}P_{i}, x^{*}(x)< 0 \mbox{ and } \forall x\in P_{i}, x^{*}(x)\geq0\bigr\} . $$

By (iii), (iv) and Lemma 2.2 of [34], for every $x_{i}^{*}\in T_{i}$, the composite functions $x_{i}^{*}\circ f_{i}$ and $x_{i}^{*}\circ g_{i}$ are continuous. For each $i\in I$, $\forall(x,y)\in X\times Y$, define:

2 $$\begin{aligned}& F_{i}(x,y)=\min\bigl\{ \bigl(x_{i}^{*} \circ f_{i}\bigr) (x,y,u_{i}):u_{i}\in S_{i}(x)\bigr\} , \end{aligned}$$

3 $$\begin{aligned}& G_{i}(x,y)=\min\bigl\{ \bigl(x_{i}^{*} \circ g_{i}\bigr) (x,y,v_{i}):v_{i}\in T_{i}(x)\bigr\} , \end{aligned}$$

4 $$\begin{aligned}& A_{i}(x,y)=\bigl\{ u_{i}\in S_{i}(x):\bigl(x_{i}^{*}\circ f_{i} \bigr) (x,y,u_{i})=F_{i}(x,y)\bigr\} , \end{aligned}$$

5 $$\begin{aligned}& B_{i}(x,y)=\bigl\{ v_{i}\in T_{i}(x):\bigl(x_{i}^{*}\circ g_{i} \bigr) (x,y,u_{i})=G_{i}(x,y)\bigr\} . \end{aligned}$$

Firstly, we show that for arbitrary $(x,y)\in X\times Y$, $A_{i}(x,y)$ and $B_{i}(x,y)$ are nonempty. In fact, $x_{i}^{*}\circ f_{i}$ and $x_{i}^{*}\circ g_{i}$ are respectively continuous on compact sets $S_{i}(x)$ and $T_{i}(x)$. Secondly, we show that $A_{i}$ is a closed mapping (similar to $B_{i}$). In fact, let $(x_{n},y_{n},u_{n})\in \operatorname{Graph}(A_{i})$ and $(x_{n},y_{n},u_{n})\rightarrow(x,y,u)\in X\times Y\times X_{i}$. Then

$$\bigl(x_{i}^{*}\circ f_{i}\bigr) (x,y,u)\leq \varliminf_{n}\bigl(x_{i}^{*}\circ f_{i}\bigr) (x_{n},y_{n},u_{n})= \varliminf_{n}F_{i}(x_{n},y_{n})\leq \varlimsup _{n}F_{i}(x_{n},y_{n})\leq F_{i}(x,y), $$

which means $(x_{i}^{*}\circ f_{i})(x,y,u)=F_{i}(x,y)$. Since $\operatorname{Graph}(S_{i})$ is closed in $X\times X_{i}$, $u_{n}\in S_{i}(x_{n})$, we obtain that $u\in S_{i}(x)$. Hence, $(x,y,u)\in \operatorname{Graph}(A_{i})$. By Lemma 3, $A_{i}$ is u.s.c. Thirdly, we show that the set $A_{i}(x,y)$ is convex. For this, let $u_{i,1}, u_{i,2}\in A_{i}(x,y)$. According to the definition of $A_{i}(x,y)$, we have $u_{i,1}, u_{i,2}\in S_{i}(x,y)$, and

6 $$ F_{i}(x,y)=x_{i}^{*}\circ f_{i}(x,y,u_{i,1})=x_{i}^{*}\circ f_{i}(x,y,u_{i,2}). $$

Let $\lambda\in(0,1)$, since $S_{i}:X\times Y\rightarrow2^{X_{i}}$ has convex values, we have $(1-\lambda)u_{i,1}+\lambda u_{i,2}\in S_{i}(x,y)$. Since the map $f_{i}(x,y,\cdot)$ is naturally quasi-convex, there exists $t\in(0,1)$ such that

$$\begin{aligned} F_{i}(x,y)&\leq x_{i}^{*}\circ f_{i}\bigl(x,y,(1-\lambda)u_{i,1}+\lambda u_{i,2} \bigr) \\ & \leq(1-t)x_{i}^{*}\circ f_{i}(x,y,u_{i,1})+t x_{i}^{*}\circ f_{i}(x,y,u_{i,2}) \\ & =(1-t)F_{i}(x,y)+t F_{i}(x,y)=F_{i}(x,y). \end{aligned}$$

That is, $x_{i}^{*}\circ f_{i}(x,y,(1-\lambda)u_{i,1}+\lambda u_{i,2})=F_{i}(x,y)$, which means $(1-\lambda)u_{i,1}+\lambda u_{i,2}\in A_{i}(x,y)$.

Assume that $L_{i}=T_{i}(X)$, $i\in I$. Since $T_{i}:X\rightarrow 2^{Y_{i}}$ is nonempty convex-valued, $L_{i}$ are nonempty convex subsets of $F_{i}$ and $L=\prod_{i\in I}L_{i}$ is a nonempty convex subset of $F=\prod_{i\in I}F_{i}$. Since $E_{i}$ is a locally convex topological vector space, $X_{i}$ is a nonempty convex subset of $E_{i}$. It is similar to knowing that $X=\prod _{i\in I}X_{i}$ is a nonempty convex subset of $E=\prod_{i\in I}E_{i}$.

Define set-valued mappings $H_{i}:X\times L\rightarrow 2^{X_{i}\times L_{i}}$, $i\in I$ as

$$H_{i}(x,y)=\bigl(A_{i}(x,y),B_{i}(x,y)\bigr),\quad \forall (x,y)\in X\times L. $$

According to the proof above, we obtain that *X* and *L* are nonempty convex. Define $H:X\times L\rightarrow2^{X\times L}$ as $H(x,y)=\prod_{i\in I}H_{i}(x,y)$. Obviously, *H* is a u.s.c. set-valued mapping with convex and compact values. By Lemma 2, there exists $(\bar{x},\bar{y})\in X\times L$ such that $(\bar{x},\bar{y})\in H(\bar{x},\bar{y})$. Thus, $\bar{x}_{i}\in S_{i}(\bar{x})$, $\bar{y}_{i}\in T_{i}(\bar{x})$ with $\bar{x}_{i}\in A_{i}(\bar{x},\bar{y})$ and $\bar{y}_{i}\in B_{i}(\bar{x},\bar{y})$. According to (4) and (5), it means that for each $i\in I$, $\bar{x}_{i}\in S_{i}(\bar{x})$, $\bar{y}_{i}\in T_{i}(\bar{x})$ such that

$$\begin{aligned}& \bigl(x_{i}^{*}\circ f_{i}\bigr) (\bar{x}, \bar{y},x_{i})\geq\bigl(x_{i}^{*}\circ f_{i}\bigr) (\bar{x},\bar{y},\bar{x}_{i}), \quad \forall x_{i}\in S_{i}(\bar{x}), \\& \bigl(x_{i}^{*}\circ g_{i}\bigr) (\bar{x}, \bar{y},y_{i})\geq\bigl(x_{i}^{*}\circ g_{i}\bigr) (\bar{x},\bar{y},\bar{y}_{i}), \quad \forall y_{i}\in T_{i}(\bar{x}). \end{aligned}$$

By conditions (iii)(a), (iv)(a), we have

$$\begin{aligned}& \bigl(x_{i}^{*}\circ f_{i}\bigr) (\bar{x}, \bar{y},x_{i})\geq0, \quad \forall x_{i}\in S_{i}(\bar{x}), \\& \bigl(x_{i}^{*}\circ g_{i}\bigr) (\bar{x}, \bar{y},y_{i})\geq0, \quad \forall y_{i}\in T_{i}(\bar{x}). \end{aligned}$$

By Lemma 1, we obtain that

$$\begin{aligned}& f_{i}(\bar{x},\bar{y},x_{i})\in P_{i}\subset C_{i}(\bar{x}),\quad \forall x_{i}\in S_{i}(\bar{x}), \\& g_{i}(\bar{x},\bar{y},y_{i})\in P_{i}\subset C_{i} (\bar{x}), \quad \forall y_{i}\in T_{i}( \bar{x}). \end{aligned}$$

Then the (SSGVQEP) has a solution. □

#### Remark 1

The following example is given to show that Theorem 1 improves [1], Theorem 3.1.

#### Example 1

For each $i\in I$, $E_{i}=F_{i}=\mathbb{R}$ and $Z_{i}=\mathbb{R}^{2}$, $X_{i}=Y_{i}=[0,1]$. Let $X=\prod _{i\in I}X_{i}$ and $Y=\prod _{i\in I}Y_{i}$. For each $i\in I$, the set-valued mappings $S_{i}:X \rightarrow2^{X_{i}}$ and $T_{i}:Y\rightarrow2^{Y_{i}}$ are defined as $S_{i}(x)=T_{i}(x)=[0,1]$. For all $(x, y, u_{i})\in X\times Y\times X_{i}$, let

$$f_{i}(x,y,u_{i})=\bigl(u_{i}^{2},1-u_{i}^{2} \bigr) $$

and for all $(x,y,v_{i})\in X\times Y\times Y_{i}$,

$$g_{i}(x,y,v_{i})=(0,0). $$

Then the assumptions of Theorem 1 hold. But the vector-valued mapping $f_{i}$ is not a properly quasi-convex mapping, and thus this example does not satisfy all the conditions of Theorem 3.1 in [1].

### Hadamard well-posedness of (SSGVQEP)

In this subsection, we will introduce Hadamard-type well-posedness for (SSGVQEP) and establish sufficient conditions of Hadamard-type well-posedness for (SSGVQEP). Broadly speaking, we say that a problem is Hadamard well-posed if it is possible to obtain ‘small’ changes in the solutions in correspondence to ‘small’ changes in the data. More precisely, let us recall the notions of Hadamard well-posedness and generalized Hadamard well-posedness.

Assume that *Z* is a metric space, the excess of the set $A\subset Z$ to the set $B\subset Z$ is defined by

7 $$ e(A,B)= \sup\bigl\{ d(a,B): a\in A \bigr\} , $$

and the Hausdorff distance between *A* and *B* is defined as

8 $$ h(A,B)= \max\bigl\{ e(A,B), e(B,A)\bigr\} . $$

For convenience, in what follows, assume that $P_{0}$ is a set of problems of (SSGVQEP) and $p_{n}$ ($n=1,2,\ldots$) means a sequence of problems of (SSGVQEP) which belong to $P_{0}$. We show that the formula of $p_{n}$ is as follows: find $(x^{n},y^{n})\in X\times Y$ such that $\forall i\in I$, $x^{n}_{i}\in S^{n}_{i}(x^{n})$, $y^{n}_{i}\in T^{n}_{i}(x^{n})$,

$$\begin{aligned}& f^{n}_{i}\bigl(x^{n},y^{n},u^{n}_{i} \bigr)\in C^{n}_{i}\bigl(x^{n}\bigr), \quad \forall u^{n}_{i}\in S^{n}_{i} \bigl(x^{n}\bigr), \\& g^{n}_{i}\bigl(x^{n}, y^{n},v^{n}_{i} \bigr)\in C^{n}_{i}\bigl(x^{n}\bigr), \quad \forall v^{n}_{i}\in T^{n}_{i} \bigl(x^{n}\bigr). \end{aligned}$$

Meanwhile, for any problem $p\in P_{0}$, the formula of *p* is showed as follows: find $(x,y)\in X\times Y$ such that $\forall i\in I$, $x_{i}\in S_{i}(x)$, $y_{i}\in T_{i}(y)$,

$$\begin{aligned}& f_{i}(x,y,u_{i})\in C_{i}(x), \quad \forall u_{i}\in S_{i}(x), \\& g_{i}(x,y,v_{i})\in C_{i}(x), \quad \forall v_{i}\in T_{i}(x). \end{aligned}$$

Given a set $P_{0}$ of (SSGVQEP), let us define the distance function $d_{P_{0}}$ as follows:

$$\begin{aligned} d_{P_{0}}(p_{1},p_{2}) =&\sup_{(x,y,u_{i})\in X\times Y\times X_{i}} \sum_{i=1}^{n}\bigl\Vert f_{i}^{1}(x,y,u_{i})-f_{i}^{2}(x,y,u_{i}) \bigr\Vert \\ &\quad{} +\sup_{(x,y,v_{i})\in X\times Y\times X_{i}}\sum_{i=1}^{n} \bigl\Vert g_{i}^{1}(x,y,v_{i})-g_{i}^{2}(x,y,v_{i}) \bigr\Vert \\ &\quad{} +\sup_{x\in X}\sum_{i=1}^{n}h \bigl(S_{i}^{1}(x),S_{i}^{2}(x)\bigr)+ \sup_{y\in Y}\sum_{i=1}^{n}h \bigl(T_{i}^{1}(x),T_{i}^{2}(x)\bigr), \end{aligned}$$

where $p_{1}=(f_{1}^{1},f_{2}^{1},\ldots,f_{N}^{1},g_{1}^{1},g_{2}^{1},\ldots ,g_{N}^{1},S_{1}^{1},S_{2}^{1},\ldots ,S_{N}^{1},T_{1}^{1},T_{2}^{1},\ldots ,T_{N}^{1})$, $p_{2}=(f_{1}^{2},f_{2}^{2},\ldots, f_{N}^{2},g_{1}^{2},g_{2}^{2}, \ldots ,g_{N}^{2},S_{1}^{2},S_{2}^{2},\ldots ,S_{N}^{2},T_{1}^{2},T_{2}^{2},\ldots,T_{N}^{2})\in P_{0}$. Let

$$\sup_{(x,y,u_{i})\in X\times Y\times X_{i}}\sum_{i=1}^{n} \bigl\Vert f_{i}(x,y,u_{i})\bigr\Vert < +\infty. $$

Clearly, $(P_{0}, d_{P_{0}})$ is a metric space.

We say that $p_{n}\rightarrow p$ if $d_{P_{0}}(p_{n},p)\rightarrow0$. Moveover, let $\Gamma(p)$ be the set of solutions of $p\in P_{0}$. Γ is a set-valued mapping from $P_{0}$ to $2^{X\times Y}$, and it is called the solution mapping of *p*.

#### Definition 3

Let $(P_{0}, d_{P_{0}})$ be the metric space of data of (SSGVQEP) problems mentioned above, let $(X\times Y, d_{X\times Y})$ be the metric space for the solutions of a problem *p* in $(P_{0}, d_{P_{0}})$ and Γ be the solution mapping from the space $(P_{0}, d_{P_{0}})$ of problems to the space $2^{X\times Y}$ of all non-empty solution subsets in $(X\times Y, d_{X\times Y})$.

1. Let $p_{n}\rightarrow p$. A problem $p\in P$ is called Hadamard well-posed (in short, *H*-*wp*) with respect to $(P_{0}, d_{P_{0}})$ and $(X\times Y, d_{X\times Y})$ if the set $\Gamma(p)$ of solutions of *p* is a singleton and any sequence $x_{n}\in\Gamma(p_{n})$ converges to the unique solution of *p*.
2. Let $p_{n}\rightarrow p$. A problem $p\in P$ is called generalized Hadamard well-posed (in short, *gH*-*wp*) with respect to $(P_{0}, d_{P_{0}})$ and $(X\times Y, d_{X\times Y})$ if the set $\Gamma(p)$ of solutions of *p* is nonempty, and any sequence $x_{n}\in\Gamma(p_{n})$ has a subsequence converging to some solution in $\Gamma(p)$.

#### Example 2

Let $I=\{ 1, 2\} $ for each $i\in I$, $E_{i}=F_{i}=\mathbb{R}$ and $Z_{i}=\mathbb{R}$, $X_{i}=Y_{i}=[0,1]$. Assume that the problem *p* is defined by $S_{i}(x)=(-1,1)$, $T_{i}(x)=\{0\}$, $C_{i}(x)=\mathbb{R}_{+}$, $f_{i}(x,y,u_{i})=x_{i}-u_{i}$ and $g_{i}(x,y,v_{i})=0$ for every $i\in I$. Define a sequence of problems $\{p_{n}\}$ by $S^{n}_{i}(x)=[-1+\frac{1}{n},1-\frac{1}{n}]$, $T^{n}_{i}(x)=\{0\}$, $C^{n}_{i}(x)=\mathbb{R}_{+}$, $f^{n}_{i}(x,y,u_{i})=x_{i}-u_{i}+\frac{1}{n}$ and $g^{n}_{i}(x,y,v_{i})=0$ for every $i\in I$. It is clear that $d(p,p_{n})\rightarrow0$, the solution set $\Gamma(p_{n})$ of $p_{n}$ is $[1-\frac{1}{2n},1-\frac{1}{n}]\times[1-\frac{1}{2n},1-\frac {1}{n}]\times\{0\}\times\{0\}$, but the problem *p* has not any solution. Therefore, the problem *p* is not Hadamard well-posed.

#### Lemma 4

*Let* $I=\{1,2,\ldots,n\}$ *be a finite set*. *For each* $i\in I$, $E_{i}$, $F_{i}$ *and* $Z_{i}$ *are metric spaces*. *Let* $X_{i}\subseteq E_{i}$ *and* $Y_{i}\subseteq F_{i}$ *be compact convex subsets and* $X=\prod _{i\in I}X_{i}$ *and* $Y=\prod _{i\in I}Y_{i}$. *Assume that the set* $\Gamma(p)$ *of solutions of* $p\in P_{0}$ *is nonempty and the following conditions are satisfied*: *for each* $i\in I$,

(i) *the set*-*valued mappings* $S_{i}:X \rightarrow2^{X_{i}}$ *and* $T_{i}:X\rightarrow2^{Y_{i}}$ *are compact closed continuous mappings with nonempty convex values*,
(ii) *the vector*-*valued mappings* $f_{i}:X\times Y\times X_{i}\rightarrow Z_{i}$ *and* $g_{i}:X\times Y\times Y_{i}\rightarrow Z_{i}$ *are continuous*.

*Then* $\Gamma(p):P_{0}\rightarrow2^{X\times Y}$ *is u*.*s*.*c*.

#### Proof

Since $X\times Y$ is compact, by Lemma 3, we need only to show that Γ is a closed mapping, i.e., to show that for any $p_{n}\in P$, $m=1,2,3,\ldots$ with $p_{n}\rightarrow p$, and for any $(x^{n},y^{n})\in\Gamma(p_{n})$ with $(x^{n},y^{n})\rightarrow(x,y)$, we have $(x,y)\in\Gamma(p)$. Since $(x^{n},y^{n})\in\Gamma(p_{n})$, we obtain $x^{n}_{i}\in S^{n}_{i}(x^{n})$ and $y^{n}_{i}\in T^{n}_{i}(y^{n})$. For any $i\in I$, by the continuity of $S_{i}$, $T_{i}$ and $p_{n}\rightarrow p$, we have $x_{i}\in S_{i}(x)$ and $y_{i}\in T_{i}(y)$. Therefore, to prove $(x,y)\in\Gamma(p)$, we only need to prove

9 $$ \begin{gathered} f_{i}(x,y,u_{i}) \in C(x_{i}),\quad \forall u_{i}\in S_{i}(x), \\ g_{i}(x,y,v_{i})\in C(x_{i}),\quad \forall v_{i}\in T_{i}(x). \end{gathered} $$

Suppose that (9) is not true, we have

$$\begin{aligned}& \exists u_{i}\in S_{i}(x), \quad \mbox{s.t.}\quad f_{i}(x,y,u_{i})\notin C(x_{i}), \\& \mbox{or} \quad \exists v_{i}\in T_{i}(x), \quad \mbox{s.t.} \quad g_{i}(x,y,v_{i}) \notin C(x_{i}). \end{aligned}$$

Without loss of generality, we assume that $\exists u_{i}\in S_{i}(x)$, *s.t.* $f_{i}(x,y,u_{i})\notin C(x_{i})$. Thus, there exists some open neighborhood *V* of the zero element of $Z_{i}$ such that

$$\bigl(f_{i}(x,y,u_{i})+V\bigr)\cap C_{i}(x)= \emptyset. $$

Since $p_{n}\rightarrow p$, there exists $n_{1}\in Z^{+}$ ($Z^{+}$ is a set of positive integers) such that when $n\geq n_{1}$, we have

10 $$\begin{aligned} f^{n}_{i}\bigl(x^{n},y^{n},u^{n}_{i} \bigr)-f_{i}\bigl(x^{n},y^{n},u^{n}_{i} \bigr)\in\frac{1}{2}V. \end{aligned}$$

Since $u_{i}\in S_{i}(x)$, $(x^{n},y^{n})\rightarrow(x,y)$ and $S_{i}$ is a compact continuous mapping, we have that there exists $u^{n}_{i}\in S^{n}_{i}(\bar{x}^{n})$ such that $u^{n}_{i}\rightarrow u_{i}$. Since $f_{i}$ is continuous at $(x,y,u_{i})$, there exists $n_{2}\in Z^{+}$ such that for any $n\geq n_{2}$, we have

11 $$\begin{aligned} f_{i}\bigl(x^{n},y^{n},u^{n}_{i} \bigr)\in f_{i}(x,y,u_{i})+\frac{1}{2}V. \end{aligned}$$

Let $N=\max\{n_{1},n_{2}\}$. By (10) and (11), we obtain that for any $n\geq N$,

$$\begin{aligned} & f^{n}_{i}\bigl(x^{n},y^{n},u^{n}_{i} \bigr)=\bigl(f^{n}_{i}\bigl(x^{n},y^{n},u^{n}_{i} \bigr)-f_{i}\bigl(x^{n},y^{n},u^{n}_{i} \bigr)\bigr)+f_{i}\bigl(x^{n},y^{n},u^{n}_{i} \bigr) \\ &\quad \in \frac{1}{2}V+\biggl(f_{i}(x,y,u_{i})+ \frac{1}{2}V\biggr)\subset\bigl(f_{i}(x,y,u_{i})+V \bigr). \end{aligned} \ $$

Since $(f_{i}(x,y,u_{i})+V)\cap C_{i}(x)=\emptyset$, we have $f^{n}_{i}(x^{n},y^{n},u^{n}_{i})\notin C_{i}(x)$, which contradicts $(x^{n},y^{n})\in\Gamma(p_{n})$. Therefore, Γ is a closed mapping. □

Now we establish the sufficient condition of Hadamard-type well-posedness for (SSGVQEP).

#### Theorem 2

*Let* $I=\{1,2,\ldots,n\}$ *be a finite set*, *for each* $i\in I$, *let* $E_{i}$, $F_{i}$ *and* $Z_{i}$ *be metric spaces*, *and* $X_{i}\subseteq E_{i}$ *and* $Y_{i}\subseteq F_{i}$ *be compact convex subsets*. *Let* $X=\prod _{i\in I}X_{i}$ *and* $Y=\prod _{i\in I}Y_{i}$. *Assume that the set* $\Gamma(p)$ *of solutions of* $p\in P_{0}$ *is nonempty and the following conditions are satisfied*: *for each* $i\in I$,

(i) *the set*-*valued mappings* $S_{i}:X \rightarrow2^{X_{i}}$ *and* $T_{i}:X\rightarrow2^{Y_{i}}$ *are compact closed continuous mappings with nonempty convex values*,
(ii) *the vector*-*valued mappings* $f_{i}:X\times Y\times X_{i}\rightarrow Z_{i}$ *and* $g_{i}:X\times Y\times Y_{i}\rightarrow Z_{i}$ *are continuous*.

*Then the problem* (*SSGVQEP*) *is generalized Hadamard well*-*posed*.

#### Proof

By Lemma 4 and Theorem 2.1 of [35], the conclusion naturally holds. □

#### Remark 2

It is easy to verify that if (SSGVQEP) has a unique solution, then the fact that (SSGVQEP) is generalized Hadamard well-posed implies that (SSGVQEP) is Hadamard well-posed.

## Conclusions

Under some weaker conditions, we have established an existence result for the solution set of a system of simultaneous generalized vector quasi-equilibrium problems, and it improved the relevant Theorem 3.1 in the work of Ansari et al. [1]. We have defined a new concept of Hadamard-type well-posedness for (SSGVQEP) and established sufficient conditions for Hadamard well-posedness of (SSGVQEP).
